# COVID-19 vaccination shifts neutrophils toward a mixed activated and regulatory phenotype in patients with severe disease

**DOI:** 10.1007/s11033-026-11778-y

**Published:** 2026-04-11

**Authors:** Yrna Lorena M. de Oliveira, Ayane de S. Resende, Mariana N. F. de Franca, Camilla Natália O. Santos, Lucas S. Magalhães, Cristiane B. Correa, Priscila L. dos S. Almeida, Angela Maria da Silva, Michael W. Lipscomb, Tatiana R. de Moura

**Affiliations:** 1 Health Sciences Graduate Program, Federal University of Sergipe, Aracaju, Sergipe 49060-100 Brazil; 2 Laboratory of Immunology and Molecular Biology, Federal University of Sergipe, Aracaju, Sergipe 49060-108 Brazil; 3 Federal Institute of Education, Science and Technology Baiano – Campus Itaberaba, Itaberaba, Bahia 46880-000 Brazil; 4 Department of Parasitology and Pathology, ICBS, Federal University of Alagoas, Maceió, Alagoas 57072-900 Brazil; 5 Physiological Sciences Graduate Program, Federal University of Sergipe, São Cristóvão, Sergipe 49100-000 Brazil; 6 Department of Pharmacology, University of Minnesota, Minneapolis, MN USA; 7 Departamento de Medicina, Hospital Universitário, Rua Claudio Batista s/n, Bairro Cidade Nova, Aracaju, Sergipe 49060-108 Brazil

**Keywords:** SARS-CoV-2, Neutrophils, Innate immunity, TREM-1

## Abstract

**Background:**

Dysregulation of the innate immune response to SARS-CoV-2 has been linked to poor outcomes in COVID-19. Neutrophils are key players in this response, displaying distinct functional profiles associated with disease severity. This study investigates how neutrophil phenotypes, and their mediators are modulated in severe COVID-19 following vaccination.

**Methods and Results:**

We conducted an observational case-control study using clinical data, serum samples, and circulating neutrophils from patients hospitalized with severe COVID-19. Neutrophils from vaccinated patients exhibited increased expression of surface markers including TREM-1, CD182, HLA-DR, and PD-L1, alongside higher HLA-DR mean fluorescence intensity (MFI). These cells also showed a higher proportion of inflammatory (CD16⁺CD182⁺TREM-1⁺) and immunoregulatory (HLA-DR⁺PD-L1⁺) subsets compared to non-vaccinated individuals. Exploratory principal component analysis (PCA) revealed a trend toward separation of vaccinated and non-vaccinated groups, suggestively driven by inflammatory cytokines (IL-6, TNF-α, GM-CSF, IL-18) and neutrophil surface markers (HLA-DR, PD-L1, TREM-1, CD16).

**Conclusions:**

These findings suggest that prior COVID-19 vaccination is associated with a distinct neutrophil activation profile in patients with severe disease, characterized by the concomitant expression of pro-inflammatory and immunoregulatory markers. This immune phenotype may reflect a more balanced inflammatory response during severe SARS-CoV-2 infection. These findings open avenues for future studies incorporating functional assays and larger, independent cohorts to confirm and extend the biological and clinical relevance of these observations.

**Supplementary Information:**

The online version contains supplementary material available at 10.1007/s11033-026-11778-y.

## Introduction

Coronavirus disease 2019 (COVID-19), caused by severe acute respiratory syndrome virus 2 (SARS-CoV-2), has resulted in a pandemic with high morbidity and mortality rates worldwide. However, since the introduction of vaccination in mid-December 2020, these high rates have been reduced [1]. Although most patients present with the disease in an asymptomatic to mild form, others manifest the severe form of the disease due to respiratory discomfort and a disproportionate immune response that can cause high levels of circulating cytokines and excessive inflammation [2–5].

Some studies have shown a heterogeneity in the immune response in severe COVID-19, which can result in a dysregulation of this system and cause tissue damage, with neutrophils being able to exhibit hyperinflammatory and/or immunosuppressive phenotypes [3, 6]. Clinical evidence has already shown that a higher number of peripheral neutrophils and a high neutrophil to lymphocyte are related to more severe outcomes in COVID-19 patients, thus indicating the immunopathological role of these innate immune cells during SARS-CoV-2 infection [7–9]. Musich et al. [10] observed that vaccination can induce a dysfunctional immune response of neutrophils, thus contributing to the activation of the adaptive immune response with the activation of B lymphocytes in the production of antibodies.

In that way, the present study aims to evaluate the phenotypic and functional profile of neutrophils in the face of SARS-CoV-2 infection after vaccination. We hypothesized that, among ICU patients with severe COVID‑19, those who had received COVID‑19 vaccination would display a neutrophil profile indicative of better immune regulation, characterized by higher expression of activation/immune‑checkpoint markers (HLA‑DR, PD‑L1) and migratory receptors (CD182, TREM‑1), together with lower systemic IL‑6, compared with unvaccinated patients.

## Materials and methods

### Study design

This is a cross-sectional study analyzing the phenotype of neutrophils obtained from hospitalized patients with or without severe COVID-19 vaccination. Patients over 20 years of age diagnosed with pneumonia associated with COVID-19, confirmed by a positive RT-qPCR for SARS-CoV-2, were included. In addition, the patients were being treated in an intensive care unit (ICU) requiring invasive oxygen support and using corticosteroid therapy. Severe condition was confirmed according to WHO technical guidance for COVID-19 (https://apps.who.int/iris/handle/10665/330854). These patients were recruited from two public referral hospitals located in Aracaju (Sergipe), Brazil, and two groups were determined: Unvaccinated patients, recruited between September 8, 2020 and December 10, 2020, patients infected before vaccination implantation; while the Vaccinated group comprised patients recruited from September 6, 2021, to January 2022, following the emergence of variants of concern and the rollout of COVID-19 vaccination [11].

The exclusion criteria were patients with any type of cancer or autoimmune disease, pregnant women and individuals who had problems during the flow cytometry experiments, such as low cell yields, reading errors and problems with antibody labeling. The use of corticosteroids was not an exclusion criterion, as this treatment was universally applied as standard care in the ICU setting for both groups.

### Clinical variables

Demographic, clinical and laboratory data were obtained from the medical records of patients hospitalized on the same date as the blood collection. The variables collected were age, gender, comorbidities, absolute leukogram values (neutrophils, eosinophils, basophils, monocytes and lymphocytes), blood count (red blood cells, hemoglobin, hematocrit and platelets), renal markers (serum urea and creatinine) and vaccination status. The clinical outcome (hospital discharge or death) was also assessed; however, some patients had missing data. The neutrophil-to-lymphocyte ratio (NLR) was calculated by dividing the absolute neutrophil count by the absolute lymphocyte count (Table 1).

### Blood collection, neutrophil obtention and serological analysis

A total of 12 mL of venous blood was collected from all patients admitted to the ICU, onde day after confirmation of SARS-CoV-2 by RT-qPCR. The samples were freshly used for the flow cytometry experiments or obtained sera were stored at –80 °C until analysis. To obtain neutrophils and make flow cytometry analysis, a protocol like previous published study was performed [12, 13].

To extract immune cells, fresh EDTA blood was diluted in a 1:1 ratio with saline and then centrifuged (400 g, 35 min, 25 °C, without braking and forced acceleration) with Ficoll-Paque PLUS™ (GE Healthcare, USA). After centrifugation, two clouds were detectable: the first consisting of mononuclear cells (PBMC) and the second band enriched with granulocytes or polymorphonuclear cells (PMN). For the purposes of this study, only the polymorphonuclear cloud was used; these were carefully collected and transferred to new tubes. These granulocytic cells then underwent two or three stages of hemolysis in order to reduce the proportion of red blood cells contained in this sample, and were then washed with saline solution and centrifuged at 400 g for 10 min, 15 °C, with a brake and acceleration of 7 (Eppendorf Centrifuge 5804R).

Subsequently, these cells were incubated with Fc block, a solution for blocking nonspecific Fc fractions, composed of 2% goat fetal serum and 2% fetal bovine serum, in order to prevent nonspecific cell staining. Next, the cells were counted under a microscope and plated at a density of 1 × 10⁶ cells per well in a 96-well plate for subsequent surface receptor staining.

The antibodies used to flow cytometry experiments were: CD11b APC; CD16 FITC; CD182 PerCP-eFluor 710; CD274 PE, HLA-DR Alexa Fluor 700, TREM-1 PE. The samples were acquired on the Attune™ NxT Flow Cytometer using version 2.4 software (Life Technologies, CA, USA). The data was analyzed using FlowJo software version V10 (Treestar Inc, Ashland, USA).

The Supplementary Fig. 1 illustrates the step-by-step process from neutrophil obtention, and the strategies used to select the cell populations. The CD11b^+^ CD16^+^ markers were used to select neutrophils region [14].

The levels of soluble TREM-1 (sTREM-1, R&D systems, Minneapolis, USA) and interleukin 6 (IL-6, Invitrogen, ThermoFisher, Waltham, USA) were assessed in serum following the manufacturers’ instructions. The cytokines: GM-CSF, IFN-γ, IL-1β, IL-2, IL-4, IL-5, IL-6, IL-12p70, IL-13, IL-18, and TNF-α were quantified in the serum using a multiplex kit ProcartaPlex™ Panel kit (Th1/Th2 Cytokine 11 Plex-ProcartaPlex™) (ThermoFisher Scientific, MA, USA).

### Statistical analysis

Firstly, all the data was tested for normality using the Shapiro-Wilk test. Fisher’s exact test was then applied to investigate differences in the characterization variables between groups. The Mann-Whitney test was used to analyze continuous variables. Spearman’s rank correlation coefficient (r) was used to assess the existence of a correlation between data. Statistical significance was defined as *p* < 0.05 (α = 0.05). All statistical tests were two-tailed unless otherwise specified. The statistical analyses and figures were developed using GraphPad Prism software version 9.4 (San Diego, USA) and in R Studio version 4.4.1. The principal component analysis (PCA) was performed and plotted using the ‘ggfortify’ package.

## Results and discussion

COVID-19 is a complex infectious disease primarily characterized by immune dysregulation. It is well established that severe diseases often develop in patients with pre-existing comorbidities linked to dysfunctional inflammatory responses [15]. All COVID-19 patients included in this study were admitted to the intensive care unit (ICU), reflecting a cohort with severe disease. Clinical and laboratory characteristics are summarized in Table 1. A total of 29 patients with laboratory-confirmed COVID-19 were enrolled, including 17 non-vaccinated (N-VAC) and 12 vaccinated (VAC) individuals. Mean age and the prevalence of major comorbidities, such as obesity, diabetes mellitus, and systemic arterial hypertension, were comparable between groups, indicating similar baseline clinical risk profiles.

**Table 1:** Characterization of the patients with severe COVID-19 patients according to vaccination status

Variable	Unvaccinated (*N*-VAC = 17)	Vaccinated (VAC = 12)	*P*	Normality reference
**General characteristics**				
Age (mean ± SD)	56.7 ± 22.77	58.25 ± 16.27	0.836^a^	´-
Sex (%)				
Female	8 (47.1)	8 (66.7)	0.451^b^	-
Male	9 (52.9)	4 (33.3)		-
Obesity (%)				
Yes	2 (11.8)	4 (33.3)	0.198^b^	-
No	15 (88.2)	8 (66.7)		-
Diabetes Mellitus (%)				
Yes	11 (64.7)	7 (58.3)	0.999^b^	-
Nos	6 (35.3)	5 (41.7)		-
Systemic arterial hypertension (%)				
Yes	13 (76.5)	7 (58.3)	0.422^b^	-
No	4 (23.5)	5 (41.7)		-
Outcome^#^				
Death	2	1	0.999^b^	-
Survival^*^	10	11		-
**Laboratorial data**				
RBCs (x10^6^/uL)	5.48 ± 6.27	3.20 ± 0.52	0.565^a^	3.90–5.20
Hemoglobin (g/dL)	9.06 ± 1.16	8.99 ± 1.16	0.884^a^	11.70–15.70
Hematocrit (%)	26.38 ± 3.65	28.35 ± 4.39	0.247^a^	36.00–47.00
Platelets (x10^3^/uL)	290.27 ± 144.61	221.27 ± 131.76	0.347^a^	150.00–450.00
WBCs (x10^3^/uL)	19052.00 ± 19063.00	14612.67 ± 4579.01	0.524^a^	4.00–11.00
Neutrophils (%)	85.17 ± 7.38	83.10 ± 8.71	0.600^a^	40.00–70.00
Eosinophils (%)	2.03 ± 2.25	2.40 ± 2.82	0.561^a^	1.00–5.00
Basophils (%)	0.43 ± 0.23	0.42 ± 0.18	0.875^a^	0.00–1.00
Lymphocytes (%)	5.95 ± 4.90	9.36 ± 6.28	0.211^a^	20.00–40.00
Monocytes (%)	5.80 ± 4.80	4.40 ± 1.74	0.155^a^	2.00–12.00
N/L Ratio^$^	11.75 ± 14.93	16.49 ± 13.66	0.216^a^	-
Urea (mg/dL)	76.25 ± 36.22	91.32 ± 48.93	0.540^a^	10.00–45.00
Creatinine (mg/dL)	1.32 ± 1.26	1.35 ± 0.87	0.468^a^	0.60–1.20

Routine laboratory parameters did not differ significantly between VAC and N-VAC patients when analyzed individually. Nevertheless, both groups exhibited hallmark alterations of severe COVID-19, including reduced hemoglobin levels relative to reference values, lymphopenia, neutrophilia, and elevated urea levels, reinforcing the systemic severity of disease across the cohort. Consistent with this, our study shows that both vaccinated and unvaccinated individuals presented with severe disease and shared similar comorbidity profiles, all requiring intensive care.

Given the central role of inflammation in COVID-19 pathogenesis, circulating levels of sTREM-1 and IL-6 were evaluated. While sTREM-1 levels were similar between groups (Supplementary Fig. 1a), IL-6 concentrations were significantly lower in VAC patients compared with N-VAC individuals (Supplementary Fig. 1b; *p* = 0.0059). This finding suggests that vaccination may attenuate systemic inflammatory signaling even in critically ill patients, although causality cannot be inferred due to the observational nature of the study.

**Fig. 1:**
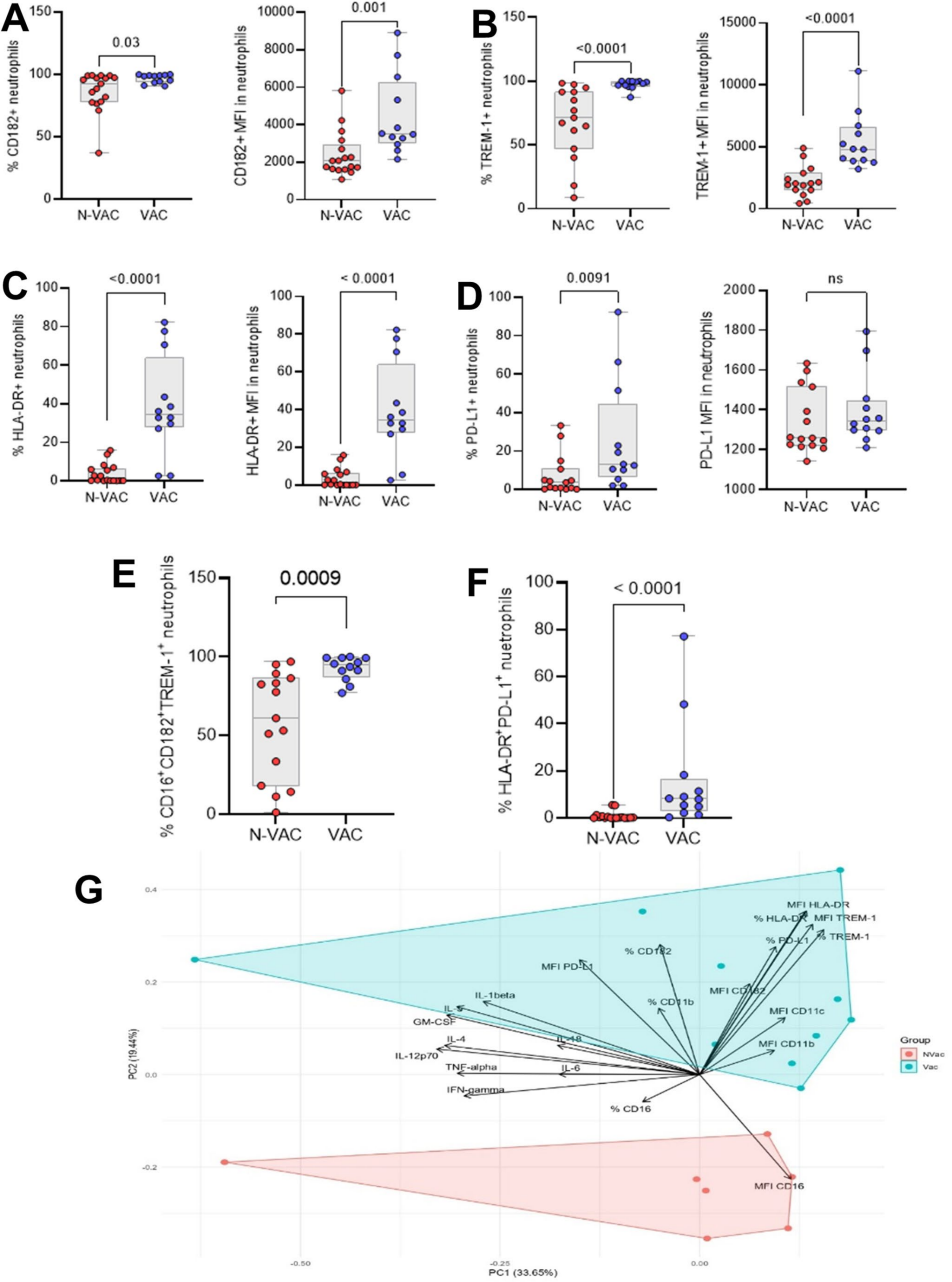
Analysis of surface receptors expressed on CD11b+CD16 + neutrophils in severe non-vaccinated and vaccinated COVID-19 patients. Percentage of cells and MFI (mean fluorescence intensity) of neutrophils expressing CD182 (**a**), TREM-1 (**b**), HLA-DR (**c**), and PD-L1 (**d**). Regulatory phenotype (**e**) and inflammatory phenotype (**f**) expression in neutrophils of severe non-vaccinated and vaccinated COVID-19 patients. Each point represents a donor. Statistical analysis performed using the Mann-Whitney test. (**g**) Evaluation of the absolute concentration of cytokines between the groups studied using principal component analysis (PCA)

To further investigate immune alterations associated with vaccination status, we focused on circulating neutrophils, a cell population known for marked phenotypic and functional plasticity in COVID-19. Phenotypic analysis of CD11b⁺CD16⁺ neutrophils revealed that VAC patients exhibited significantly higher frequencies and mean fluorescence intensities (MFI) of CD182⁺, TREM-1⁺, and HLA-DR⁺ neutrophils compared with N-VAC patients (Fig. 1a–c). In addition, PD-L1⁺ neutrophils were more abundant in the VAC group (Fig. 1d). These phenotypic differences likely reflect prior antigen exposure through vaccination and suggest that vaccination may shape neutrophil activation states during subsequent severe SARS-CoV-2 infection.

We further explored neutrophil multifunctionality by assessing co-expression patterns of surface markers. Two subsets were defined: a pro-inflammatory phenotype (CD182⁺TREM-1⁺) and an immunoregulatory phenotype (HLA-DR⁺PD-L1⁺) (Fig. 1e–f). Both subsets were significantly enriched in vaccinated patients. Importantly, although these surface marker profiles suggest differences in activation and regulatory potential, functional assays such as degranulation, phagocytosis, and neutrophil extracellular trap (NET) formation were not performed. Therefore, it remains unclear whether the observed phenotypic differences translate into functional alterations, representing a key limitation and an important direction for future investigations.

Our previous work has shown that immunological differences can be detected within the same individuals before and after vaccination, or among individuals with similar clinical manifestations but divergent immune profiles [16]. Neutrophil heterogeneity plays a critical role in the progression of COVID-19 and in shaping the immune response following vaccination. SARS-CoV-2 infection is known to increase the frequency of low-density neutrophils (LDNs), which have been associated with enhanced inflammatory activity and disease severity [17]. Furthermore, neutrophil extracellular trap (NET) formation may exacerbate tissue damage, contributing to complications such as acute respiratory distress syndrome (ARDS). In contrast, regulatory neutrophil subsets can dampen inflammation and aid tissue repair, potentially exerting a protective role in severe disease [18].

To further investigate the immune landscape, we measured a panel of 11 Th1/Th2 cytokines. Univariate analyses did not reveal statistically significant differences in absolute cytokine concentrations between VAC and N-VAC groups. However, to explore potential multivariate immune patterns, we performed a principal component analysis (PCA) as an exploratory approach (Fig. 1g). The first two principal components explained 53.09% of the total variance (PC1: 33.65%; PC2: 19.44%), revealing a trend toward separation between vaccinated (VAC) and non-vaccinated (N-VAC) patients. Inflammatory cytokines, IL-6, TNF-α, GM-CSF, and IL-18, and neutrophil markers (% HLA-DR, PD-L1, TREM-1, and CD16) were the primary drivers of this segregation.

Given the limited sample size, we emphasize that the PCA findings are hypothesis-generating rather than confirmatory. While individual cytokines did not differ significantly in univariate analyses, their combined contribution to variance in the PCA may reflect subtle immunological trends associated with vaccination status. Independent validation in larger cohorts will be necessary to confirm the robustness and biological significance of these patterns.

Notably, VAC patients clustered with neutrophils expressing higher levels of TREM-1, HLA-DR, PD-L1, and CD182, consistent with an activated phenotype potentially capable of modulating inflammation and chemotaxis. This dual expression of pro-inflammatory and immunoregulatory markers aligns with current concepts of immune adaptation in severe infections, where excessive inflammation and immune suppression may coexist. Immune dysregulation in severe infections and sepsis is characterized by a transition from hyperinflammation to immunosuppression, with similar mechanisms described for severe COVID-19 [19, 20]. These molecules are associated with neutrophil recruitment and migration to infection sites, contributing to the inflammatory process in COVID-19 [21].

We observed an activated neutrophil phenotype in vaccinated patients, marked by migratory potential and expression of molecules involved in immune regulation and co-activation of the adaptive immune response, such as HLA-DR and PD-L1. Studies indicate these molecules are involved in both protective and harmful inflammatory responses in diseases like COVID-19. For example, Seery et al. (2021) [22], in a pediatric population with multisystem inflammatory syndrome associated with COVID-19, identified a neutrophil phenotype with reduced adhesion molecule expression and increased levels of activation markers such as HLA-DR, CD64, PECAM-1, as well as inhibitory receptors including LAIR-1 and PD-L1. Similarly, Sabbatino et al. [23] reported that PD-L1 expression is associated with phenotypic variation in COVID-19 patients and may increase as part of the inflammatory process, contributing to T cell exhaustion and impaired pathogen clearance. However, distinct neutrophil phenotypes emerged between the two groups, highlighting the potential immunomodulatory effect of vaccination even in critically ill patients.

It is also important to consider that the immune response in these critically ill patients may have been further modulated by other factors, such as concomitant therapies typically administered in intensive care units, including corticosteroids, antiviral agents, and immunomodulatory drugs like tocilizumab [3–4, 6]. Moreover, the circulation of different SARS-CoV-2 variants during the study period could have influenced the observed immune profiles, since viral variants are known to differentially impact host immune responses and disease severity [11].

An additional limitation of the present study is the absence of IL-8 measurements. IL-8 is a central chemokine involved in neutrophil recruitment and activation and has been strongly associated with COVID-19 severity and neutrophil dysregulation. Due to constraints in sample volume and assay availability at the time of study design, IL-8 was not included in our cytokine panel. We acknowledge that its measurement would provide important laborator**y** insights and strongly recommend its inclusion in future studies.

Despite these limitations, our findings suggest that vaccination may influence neutrophil activation states and systemic inflammatory profiles in patients with severe COVID-19 requiring ICU admission. Specifically, vaccinated individuals exhibited lower IL-6 levels and a distinct neutrophil phenotype characterized by increased expression of CD182, TREM-1, HLA-DR, and PD-L1. Although exploratory, these results contribute to a growing body of evidence indicating that vaccination can modulate innate immune responses beyond protection against infection or severe disease. Similar immunoprofiling approaches have demonstrated the influence of prior immunological events, such as vaccination, on innate cell function in both viral infections and inflammatory diseases [24–25].

In conclusion, this study provides preliminary evidence that prior COVID-19 vaccination may shape neutrophil phenotypes and attenuate inflammatory signaling in critically ill patients. Larger, longitudinal studies incorporating functional assays and expanded cytokine profiling will be essential to validate these observations and to elucidate the mechanisms by which vaccination influences innate immune responses during severe viral infection.

## Supplementary Information

Below is the link to the electronic supplementary material.

Supplementary Material 1

## Data Availability

All data supporting the findings of this study are available within the manuscript and its supplementary materials. Additional data may be obtained from the corresponding author upon reasonable request.
