# Supplementary material for: COVID-19 vaccination shifts neutrophils toward a mixed activated and regulatory phenotype in patients with severe disease
Source: Mol Biol Rep. 2026 Apr 11;53(1):610. doi: 10.1007/s11033-026-11778-y (PMC13070084; doi:10.1007/s11033-026-11778-y)
Supplement: Supplementary file 1 — Supplementary Material 1 [file 11033_2026_11778_MOESM1_ESM.docx]

**Supplementary Material**

**COVID-19 vaccination shifts neutrophils toward a mixed activated and regulatory phenotype in patients with severe disease**

Yrna Lorena Matos de Oliveira, Ayane de Sá Resende, Mariana Nobre Faria de Franca; Camilla Natália Oliveira Santos; Lucas Sousa Magalhães; Cristiane Bani Correa; Michael Wheeler Lipscomb, and Tatiana Rodrigues de Moura

**
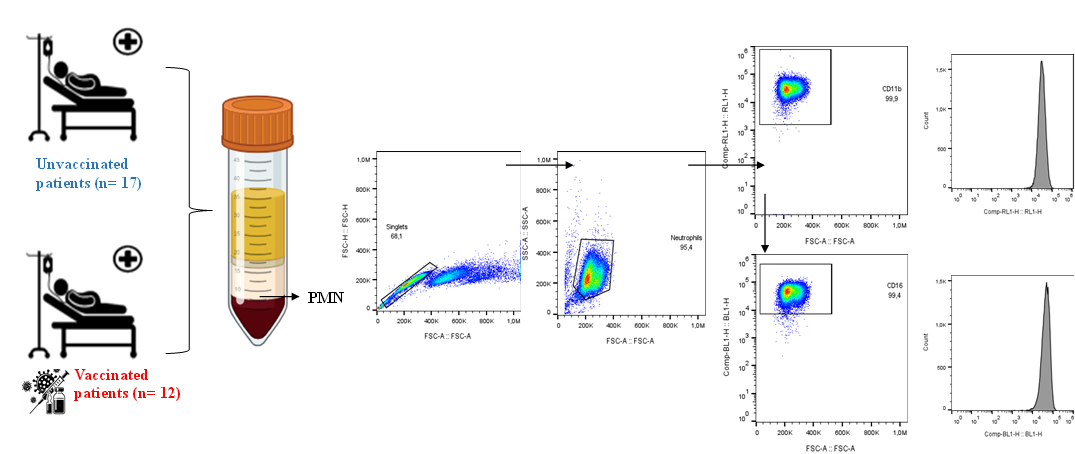
**

**Figure 1** Selection strategies for neutrophil populations. A sample of peripheral blood was collected from each group, which was then freshly directed to the steps of extracting immune cells by flow cytometry. After sample acquisition, the data was sent to Flow Jo, as indicated in the figure. Legend: PMN: polymorphonuclear cells; N: sample number; N-VAC: non-vaccinated, VAC: vaccinated.

**
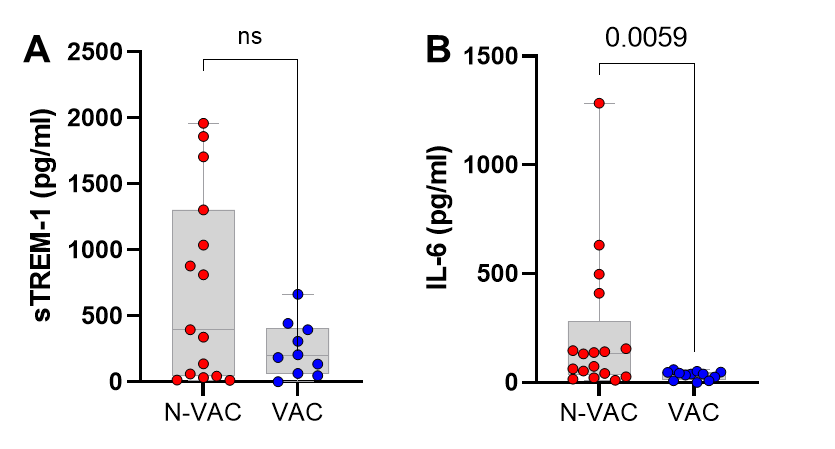
**

**Figure 2** Serum levels of sTREM-1 and IL-6 in severe non-vaccinated and vaccinated COVID-19 patients. Statistical analysis performed using the Mann-Whitney test. Each dot represents patients. N-VAC: non-vaccinated, VAC: vaccinated.
